# Anticancer Potential of Aqueous Ethanol Seed Extract of *Ziziphus mauritiana* against Cancer Cell Lines and Ehrlich Ascites Carcinoma

**DOI:** 10.1155/2011/765029

**Published:** 2010-09-26

**Authors:** Tulika Mishra, Madhu Khullar, Aruna Bhatia

**Affiliations:** ^1^Immunology and Immunotechnology Laboratory, Department of Biotechnology, Punjabi University, Patiala, Punjab 147 002, India; ^2^Experimental Medicine & Biotechnology, Post Graduate Institute of Medical Education & Research, Chandigarh 160 012, India

## Abstract

*Ziziphus mauritiana* (Lamk.) is a fruit tree that has folkloric implications against many ailments and diseases. In the present study, anticancer potential of seed extract of *Ziziphus mauritiana in vitro* against different cell lines (HL-60, Molt-4, HeLa, and normal cell line HGF) by MTT assay as well as *in vivo* against Ehrich ascites carcinoma bearing Swiss albino mice was investigated. The extract was found to markedly inhibit the proliferation of HL-60 cells. Annexin and PI binding of treated HL-60 cells indicated apoptosis induction by extract in a dose-dependent manner. The cell cycle analysis revealed a prominent increase in sub Go population at concentration of 20 *μ*g/ml and above. Agarose gel electrophoresis confirmed DNA fragmentation in HL-60 cells after 3 h incubation with extract. The extract also exhibited potent anticancer potential *in vivo*. Treatment of Ehrlich ascites carcinoma bearing Swiss albino mice with varied doses (100–800 mg/kg b.wt.) of plant extract significantly reduced tumor volume and viable tumor cell count and improved haemoglobin content, RBC count, mean survival time, tumor inhibition, and percentage life span. The enhanced antioxidant status in extract-treated animals was evident from decline in levels of lipid peroxidation and increased levels of glutathione, catalase, and superoxide dismutase.

## 1. Introduction

The plant-derived compounds have always been an important source of medicines for various diseases and have received considerable attention in recent years due to their diverse pharmacological properties including cytotoxic and cancer chemopreventive effects [1]. During the last few years, novel chemopreventive agents of natural origin have been targeted with fruits and vegetables being a key interest due to high content of bioactive compounds [2]. Cancer is the second leading cause of death all over the world [3]. According to World Health Organization, more than 10 million new cases of cancer are diagnosed every year, and the statistical trends indicate that this number would double by 2020 [4]. Cancer is the uncontrolled growth and spread of abnormal cells, associated with dysregulation of apoptosis, a programmed cell death. Most of the current anticancer drugs are derived from plant sources, which act through different pathways converging ultimately into activation of apoptosis in cancer cells leading to cell cytotoxicity. 

*Ziziphus mauritiana* commonly known as Indian jujube is a fruit tree belonging to family Rhamnaceae. Traditionally, the fruit has been used as anodyne, sedative, tonic anticancer, potent wound healer, applied on cuts and ulcers and has also been used against asthma [5, 6]. The extracts from fruits [7], leaves [8, 9], and seeds [10] of *Ziziphus mauritiana* have been reported to exhibit antioxidant activity, whereas bark [11, 12] and pulp [13] are reported to possess cytotoxicity against different cancer cell lines. Keeping above in view, the present study was aimed at investigating the effect of aqueous-ethanolic seed extract of *Ziziphus mauritiana* against different cancer cell lines *in vitro* and against Ehrlich ascites carcinoma *in vivo*.

## 2. Materials and Methods

### 2.1. Chemicals and Reagents

3-(4,5-dimethylthiazole-2-yl)-2,5-diphenyltetrazolium bromide (MTT), RPMI-1640, L-glutamine, penicillin, streptomycin, HEPES, 2-mercaptoethanol, propidium iodine (PI), DNase free RNase, proteinase K, 2-Deoxy-D-ribose, ascorbic acid, and 1,1-diphenyl-2-picrylhydrazyl (DPPH) were purchased from Sigma chemical Co, St. Louis, USA. Fetal Bovine serum (FBS) was purchased from GIBCO Invitrogen Corporation, USA. Annexin V-FITC apoptosis detection kit and Cycle test were purchased from BD Bioscience, USA. Electrophoresis apparatus and reagents were procured from Biorad, USA. Trichloro acetic acid (TCA), Thiobarbituric acid (TBA), and pyragallol were purchased from Merck, USA. All other chemicals used were of analytical grade, available locally.

### 2.2. Plant Source, Extract Preparation, and Standardization (ZMS)

Fruits of *Ziziphus mauritiana* (Lamk.), variety Umran were collected from Botanical Gardens of Punjabi University, Patiala, Punjab, India and authenticated by Professor R.C. Gupta, Botany Department, Punjabi University, Patiala, Punjab, India. Plant sample has been kept in Voucher specimen DOB (305) PUP at Punjabi University, Patiala. Aqueous-ethanolic seed extract of *Ziziphus mauritiana* (ZMS) was prepared and standardized as described earlier [14].

### 2.3. Anticancer Activity of ZMS In Vitro

#### 2.3.1. Cell Lines, Growth Conditions, and Treatment

Human promyelocytic leukemia cells (HL-60), acute lymphoblastic leukemia cells (Molt-4), human cervical cancer cells (HeLa) cells, and normal human gingival fibroblast cells (HGF) were obtained from National Cancer Institute (NCI), Bethesda, USA.

The cells were grown in RPMI-1640 medium, supplemented with 10% heat-inactivated fetal bovine serum (FBS), penicillin (100 units/mL), streptomycin (100 *μ*g/mL), HEPES 15 mM, L-glutamine (0.3 mg/mL), pyruvic acid (0.11 mg/mL), 2-mercaptoethanol (50 *μ*M), and NaHCO_3_ (0.37%) and incubated at 37°C in an atmosphere of 95% air and 5% CO_2_, with 98% humidity. ZMS was dissolved in 30% dimethyl sulfoxide (DMSO) and delivered to cell culture in complete medium while the controls received only DMSO (<0.2%, v/v).

#### 2.3.2. Cell Proliferation Assay

Cell proliferation was assessed according to the method of Shashi et al. [15]. Briefly, 200 *μ*L of medium containing 2.5 × 10^4^ cells obtained from different cell lines (HL-60, Molt-4, HeLa cells, and HGF) were seeded in each well of 96-well microtiter plates. Cells were incubated with different concentrations of ZMS for 48 hrs at 37°C, 5% CO_2_ with 98% humidity. The medium was replaced with fresh medium containing 100 *μ*g/mL of 3-(4,5-dimethylthiazole-2-yl)-2,5-diphenyltetrazolium bromide (MTT) for 4 hrs. The supernatant was aspirated and MTT-formazan crystals were dissolved in 100 *μ*L DMSO. Absorbance was measured at 570 nm. Cell growth was calculated by comparing the absorbance of treated and untreated cells. Cell line showing maximum sensitivity was subjected to further study.

#### 2.3.3. Flow Cytometric Analysis of Apoptosis and Necrosis

Flow cytometric analysis of apoptosis and necrosis was done by method of Del Bino et al. [16]. HL-60 cells treated with ZMS were washed twice with phosphate buffered saline (PBS) and then resuspended in 100 *μ*L of binding buffer provided with apoptosis detection kit (BD Pharmingin). Cells were stained with annexin-V FITC antibody and PI as per instructions provided by the manufacturer and scanned for fluorescence intensity in FL-1 (FITC) and FL-2 (PI) channels. The fractions of cell population in different quadrants were analyzed using quadrant statistics. Cells in the lower right quadrant represented apoptosis and in the upper right represented postapoptotic necrosis.

#### 2.3.4. DNA Content and Cell Cycle Phase Distribution

Cell cycle phase distribution was done by the method of Del Bino et al. [16]. Cells were treated with different concentrations of ZMS for 24 hrs and collected at 160 × g for 5 minutes in 5 mL polystyrene tubes. Cells were washed once with PBS and fixed in 70% ethanol overnight at 4°C. Cells were again washed with PBS, resuspended in 250 *μ*L PBS, incubated with RNase at 37°C, and stained with propidium iodine using procedures and reagents as described in the instruction manual. The preparations were analyzed for DNA content using BD-LSR flow cytometer. Data were collected in list mode on 10,000 events for FL2-A versus FL2-W.

#### 2.3.5. DNA Fragmentation

DNA fragmentation was assessed by electrophoresis of extracted genomic DNA from HL-60 cells as described by Muller et al. [17] with slight modifications. Briefly, 2 × 10^6^ cells were incubated with different concentrations of ZMS for 24 hours and washed with PBS containing 10 mM EDTA. The pellet was lysed in 250 *μ*L of lysis buffer (100 mM NaCl, 5 mM EDTA, 10 mM Tris-HCl, pH 8.0, 5% Triton X-100, 0.25% SDS), containing 400 *μ*g/mL DNase-free RNase and incubated at 37°C for 90 minutes followed by 1 hour incubation with proteinase-K (200 *μ*g/mL) at 50°C. DNA was extracted with 200 *μ*L of phenol : chloroform : isoamyl alcohol (25 : 24 : 1) for 1 minute and centrifuged at 13000 × g for 3 minutes. The aqueous phase was further extracted with chloroform and centrifuged. DNA was precipitated from aqueous phase with 3 volumes of chilled alcohol containing 0.3 M sodium acetate at 4°C overnight. The precipitate was centrifuged at 13000 × g for 10 minutes. DNA pellet was washed with 80% alcohol, dried, dissolved in 50 *μ*L TE buffer and electrophoresed in 1.8% agarose gel at 50 V, stained with ethidium bromide, and visualized in Bio-rad Gel Documentation System. 

For the time-dependent study, 2 × 10^6^ cells were incubated with specified concentrations of ZMS for 24 hours and studied at different time intervals. 

### 2.4. Anticancer Activity of ZMS In Vivo

#### 2.4.1. Animals

Male Swiss albino mice (18–22 g) maintained on standard laboratory diet (Kisan Feeds Ltd., Mumbai, India), having free access to tap water, were employed in the present study. The animals were housed in the departmental animal house and exposed to 12 hours cycle of light and dark. Experimental protocol was approved by Institutional Animal Ethics Committee and care of the animals was carried out as per the guidelines of Committee for the Purpose of Control and Supervision of Experiments on Animals (CPCSEAs), Ministry of Environment and Forests, Government of India (Reg. no. 107/1999/CPCSEA).

#### 2.4.2. Acute Toxicity

The acute toxicity of *Ziziphus mauritiana *seed extract was assessed as described earlier [18].

#### 2.4.3. Experimental Design

Ehrlich Ascites Carcinoma (EAC) cells maintained in the peritoneal cavity of Swiss albino mice (male) were collected from an animal having 8–10 days old ascitic tumor by aspirating the ascitic fluid in sterile isotonic saline. The viable EAC cells were counted (trypan blue indicator) under microscope. Male Swiss albino mice weighing 18–22 g were injected with 1 × 10^7^ EAC cells intraperitoneally on day 0. A day of incubation was allowed for multiplication of the cells. On day 1, the animals were randomized and divided into seven groups (*n* = 10), namely, Group I, vehicle (Distilled water, i.p); Group II, Control 5-fluorouracil (20 mg/kg b.wt.); Group III, extract (100 mg/kg b.wt.); Group IV, extract (200 mg/kg b.wt.); Group V, extract (400 mg/kg b.wt.); Group VI, extract (800 mg/kg b.wt.); Group VII, Control (1 × 10^7^ EAC cells). Test groups were treated with plant extract prepared in distilled water intraperitoneally at the interval of 24 hours from day 1 to day 9. Positive control group was treated with 5-fluorouracil (commercial anticancer drug) from day 1 to day 9, parallel control group was treated with distilled water only. The animals were sacrificed on day 13 by cervical dislocation and tumor volume, tumor cell count, hematological parameters and antioxidant status were assessed. The second set of animals was continued with the similar design so as to observe their life span.

#### 2.4.4. Tumor Growth Response

The antitumor effect of ZMS was assessed by change in body weight, ascites tumor volume, viable tumor cell count, percentage tumor inhibition, mean survival time and percentage increased life span [19, 20].

The tumor growth inhibition was calculated as follows:

(1) $$\begin{array}{cc} & \text{\%}\text{tumor}\text{inhibition}\\ & =1-\text{Av}.\text{No}.\text{of}\text{cells}\text{in}\text{test}\text{group}\\ & \;/\text{Av}.\text{No}.\text{of}\text{cells}\text{in}\text{control}\text{group}\times 100. \end{array}$$

The effect of ZMS on tumor growth was monitored by recording the mortality for the period of 40 days and percentage increase in life span was calculated as follows:

(2) $$\begin{array}{cc} & \text{\%}\text{ILS}\\ & =[(\text{Mean}\text{survival}\text{time}\text{of}\text{treated}\text{group}\;\;\\ & \;\;\;\;/\text{Mean}\text{survival}\text{time}\text{of}\text{control}\text{group}\times 100)-1],\\ & \text{Mean}\text{Survival}\\ & =\left(\text{Day}\text{of}1\text{st}\text{death}+\text{Day}\text{of}\text{last}\text{death}\right)/2. \end{array}$$

Body weight of the animals was recorded both in treated and control groups at the beginning of the experiment and subsequently on every 5th day.

#### 2.4.5. Hematological Studies

Blood was collected from retro-orbital plexus of animals and used for the estimation of hemoglobin (Hb) content, red blood cell count (RBC), and white blood cell count (WBC) [21] and animals were sacrificed by cervical dislocation.

#### 2.4.6. Biochemical Assays

The liver of animals was excised, rinsed in ice-cold normal saline followed by 0.15 M Tris-HCl (pH 7.4), blotted dry, and weighed. A 10% (w/v) homogenates was prepared in 0.15 M Tris-HCl buffer and was used for the estimation of lipid peroxidation (LPO) [22] and glutathione content (GSH) [23]. Rest of the homogenate was centrifuged at 2500 × g for 15 minutes at 4°C and superoxide dismutase (SOD) [24], catalase (CAT) [25], and total protein [26] was estimated in the supernatant.

#### 2.4.7. Statistical Analysis

All the results of *in vivo* experiment were expressed as Mean ± S.E.M. Data of tests were statistically analyzed using one-way ANOVA followed by Tukey's multiple range test, applied for *post-hoc* analysis. The data were considered to be statistically significant if the probability had a value of 0.05 or less.

## 3. Results

### 3.1. Inhibition of Cell Proliferation in Various Cell Lines by ZMS

**Figure 1:**
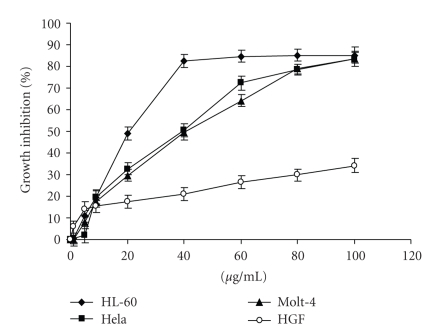
Proliferation inhibition in different human cell lines upon ZMS treatment. ZMS at indicated concentrations were evaluated for its in vitro cytotoxicity against different cancer cell lines (HL-60, HeLa, and Molt-4) and normal cell line (HGF) employing MTT reduction assay. The results are expressed as percent of cell growth inhibition determined relative to untreated control cells. Data are mean value ± S.E.M. (*n* = 8) and representative of one of the three similar experiments.

In order to determine the anticancer potency of extract *in vitro*, ZMS was tested for cell growth inhibition in different cancer cell lines at various concentrations for 48 hours. The extract did not show any significant inhibitory effect against normal cell line HGF (Normal human gingival fibroblast cell line). Inhibitory effect against HL-60, Hela, and Molt-4 was observed with IC50 value at 20 *μ*g/mL, 40 *μ*g/mL, and 40 *μ*g/mL, respectively. Of the tested cell lines, maximum growth inhibition was observed in HL-60 cells as shown in Figure 1, and thus HL-60 was selected for further study. 

### 3.2. Flow Cytometric Analysis of Apoptosis/Necrosis Induced by ZMS in HL-60 Cells

**Figure 2:**
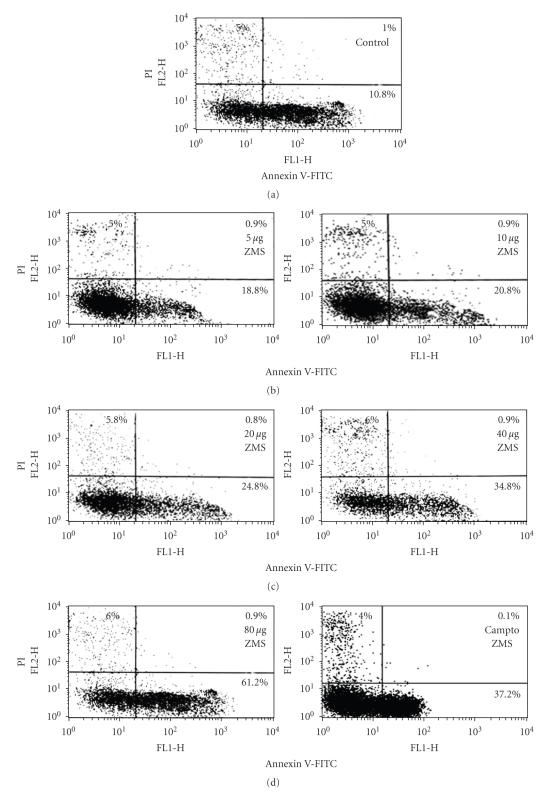
Flow cytometric analysis of ZMS-induced apoptosis and necrosis in HL-60 cells using annexinV-FITC and PI double staining. HL-60 cells (1 × 106 /mL) were incubated with indicated concentrations of ZMS for 12 hours and stained with Annexin V-FITC/PI. Quadrant analysis of fluorescence intensity of ungated cells in FL-1 versus FL-2 channels was from 10000 events. Cells in the lower right quadrant represented apoptosis while in the upper right quadrant indicated postapoptotic necrosis and representative of one of the three similar experiments.

In order to determine whether ZMS-induced cytotoxicity is due to apoptosis or necrosis, HL-60 cells were incubated with different concentrations of ZMS for 12 hours, and the percentage of cells undergoing apoptosis or necrosis was determined by staining with annexin V-FITC and PI (Figure 2). Cells in the lower right quadrant indicated apoptosis while in the upper right quadrant represented postapoptotic necrotic population. ZMS at 5 *μ*g/mL and 80 *μ*g/mL induced about 18.8% and 61.2% apoptosis. The increase in annexin V-FITC/PI positive cell population suggests that ZMS is a potent inducer of apoptosis and triggers events leading to apoptotic cell death. 

### 3.3. Cell Cycle Analysis by ZMS Treatment

**Figure 3:**
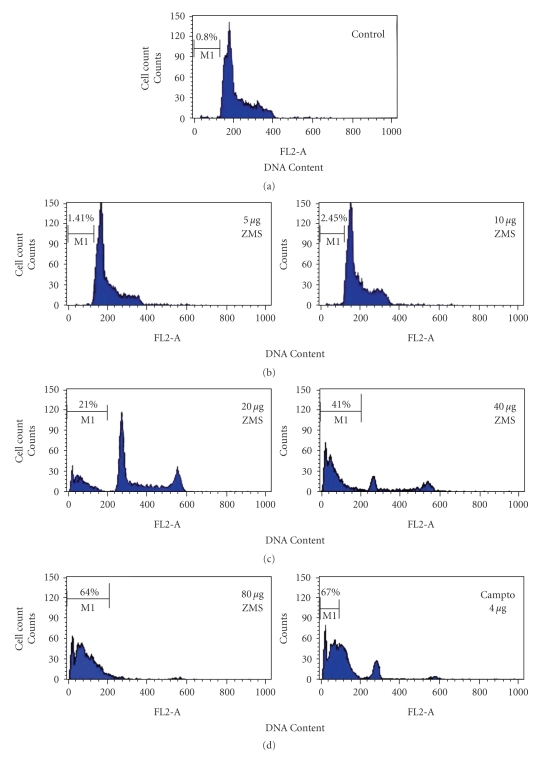
Cell cycle analysis in HL-60 cells after ZMS treatment. HL-60 cells (1 × 106 /mL) were treated with different concentration of ZMS for 24 hours. Cells were stained with PI to determine DNA fluorescence by flow cytometer. Sub-GO population indicative of DNA damage was analyzed from the hypodiploid sub-Go fraction (<2*n* DNA) of DNA cell cycle analysis. The cells for hypodiploid (sub GO/G1, <2*n* DNA) population were analyzed from FL2-A versus cell counts shown in % and representative of one of the three similar experiments.

For the confirmation of apoptotic cell death induced by ZMS in HL-60 cells, the cells were treated with ZMS for 24 hours. The cells exhibited concentration-dependent increase in hypodiploid sub-Go/G1 DNA fraction (<2nDNA) (Figure 3). The sub-Go/G1 fraction was <1% in control cells (untreated HL-60 cells) which increased with increasing concentration of ZMS and reached to 64% at concentration of 80 *μ*g/mL.

### 3.4. DNA Fragmentation

**Figure 4:**
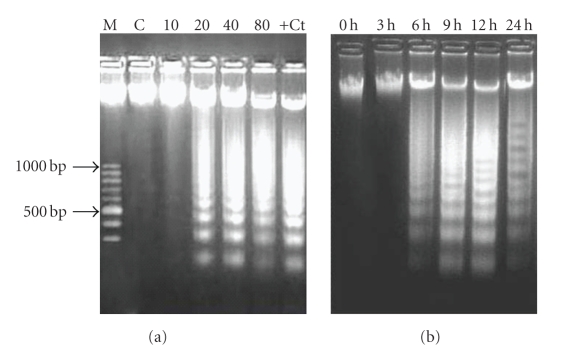
Dose-dependent and time-dependent induction of DNA fragmentation in HL-60 cells after ZMS treatment. (a) 2 × 106 HL-60 cells treated with different concentrations of ZMS extract for 24 hours. DNA was electrophoresed on 1.8% agarose gel and stained with ethidium bromide. M-Marker; C-Untreated HL-60 cells; 10-10 *μ*g/mL of ZMS treated HL-60 cells; 20-20 *μ*g/mL of ZMS treated HL-60 cells; 40-40 *μ*g/mL of ZMS treated HL-60 cells; 80-80 *μ*g/mL of ZMS treated HL-60 cells; +Ct− Camptothecin treated HL-60 cells. (b) 2 × 106 HL-60 cells were treated with 20 *μ*g/mL of ZMS extract and were incubated for 3 hours, 6 hours, 9 hours, 12 hours, and 24 hours. DNA was electrophoresed at respective time interval on 1.8% Agarose gel and stained with ethidium bromide.

DNA fragmentation, a characteristic feature of apoptosis, was assessed by ladder formation on agrose gel electrophoresis. HL-60 cells treated with extract showed apoptosis in a concentration-dependent manner as evidenced by the formation of internucleosomal DNA fragments (Figure 4(a)). Apoptosis induction in cells started at 20 *μ*g/mL and this concentration was further selected for time-dependent study. The extract was found to induce apoptosis in cells after 3 hours of incubation as shown in Figure 4(b).

### 3.5. Survival Time and Tumor Growth Response

Antitumor activity of ZMS against EAC tumor-bearing mice is shown in Table 1. The tumor volume and viable cell count significantly increased in EAC control animals. Administration of ZMS (100, 200, 400, and 800 mg/kg b.wt.) to tumor-bearing mice significantly (*P* < .001) decreased tumor volume and viable cell count and enhanced the mean survival time in a dose-dependent manner. Tumor growth inhibition upto 75.42 ± 0.96% was observed at 800 mg/kg b.wt. with 70% increase in life span of test animals.

**Figure 5:**
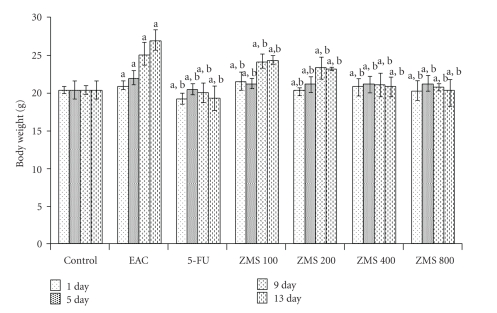
Change in body weight in EAC bearing mice after ZMS treatment. The results are presented as Mean ± S.E.M. (*n* = 10). (a) *P* < .001 in comparison to untreated control; (b) *P* < .05 in comparison to EAC control group.

EAC-bearing mice showed rise in body weight, while the extract treatment inhibited the rise in body weight in the pattern similar to the standard drug (Figure 5).

### 3.6. Hematological Parameters

Hematological parameters of tumor bearing animals on day 13 were found to be significantly altered as compared to normal group (Table 1). Hemoglobin content and RBC count in EAC control group decreased while WBC count increased as compared to normal group. Treatment with ZMS at various doses increased hemoglobin content and RBC count and reduced the WBC count towards normalcy.

### 3.7. Lipid Peroxidation and Glutathione

**Figure 6:**
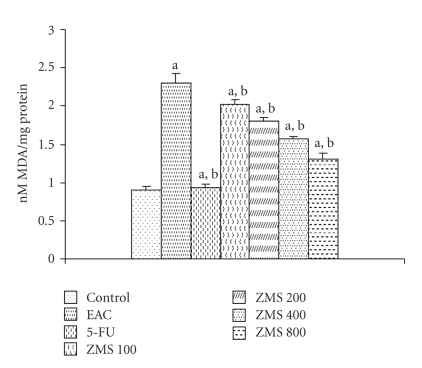
Reduction in lipid peroxidation in EAC-bearing mice after ZMS treatment. The results are presented as Mean ± S.E.M. (*n* = 10). (a) *P* < .001 in comparison to normal control; (b) *P* < .05 in comparison to EAC control group.

Increase in malondialdehyde (MDA) levels is a measure of lipid peroxidation. Figure 6 depicts the level of lipid peroxidation in terms of MDA in liver tissue of experimental animals. It was observed that MDA levels increased by 60.6% in EAC control group as compared to normal control group (*P* < .001). After administration of ZMS at different doses (100, 200, 400, and 800 mg/kg) to EAC-bearing mice, the level of lipid peroxidation reduced by 12.22%, 21.83%, 31.44%, and 43.23%, respectively, as compared to EAC control group (*P* < .05).

**Figure 7:**
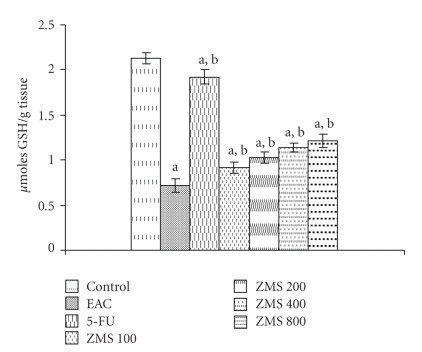
Augmentation of glutathione content in EAC bearing mice after ZMS treatment. The results are presented as Mean ± S.E.M. (*n* = 10). (a) *P* < .001 in comparison to normal control; (b) *P* < .05 in comparison to EAC control group.

Figure 7 illustrates the effect of ZMS on reduced glutathione in EAC-bearing mice. Tumor induction by inoculation of EAC drastically decreased the GSH content by 70% (*P* < .001) in control group. The administration of ZMS at 100, 200, 400 and 800 mg/kg to EAC bearing animals increased the GSH content by 21.97%, 30.39%, 37.71% and 41.32%, respectively, as compared to untreated EAC control group (*P* < .05).

### 3.8. Antioxidant Enzymes

**Figure 8:**
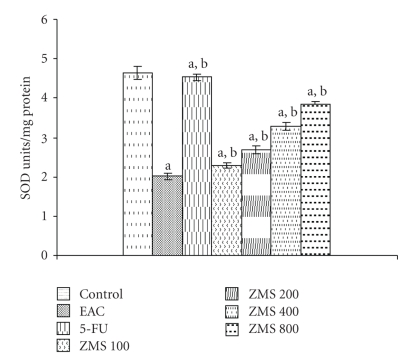
Augmentation of superoxide dismutase activity in EAC bearing mice after ZMS treatment. The results are expressed as Mean ± S.E.M. (*n* = 10). (a) *P* < .001 in comparison to normal control; (b) *P* < .05 in comparison to EAC control group.

ZMS significantly restored the antioxidant status of tumor-bearing mice. The effect of administration of ZMS to EAC bearing animals at 100, 200, 400, and 800 mg/kg efficiently enhanced the superoxide dismutase activity by 11.01%, 24.62%, 31.52%, and 47.12%, respectively, as compared to EAC control group (Figure 8). 

**Figure 9:**
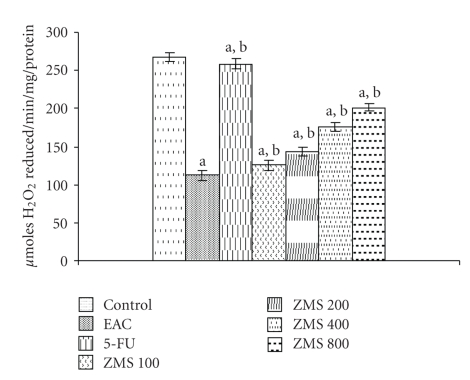
Augmentation of catalase activity in EAC bearing mice after ZMS treatment. The results are expressed as Mean ± S.E.M. (*n* = 10). (a) *P* < .001 in comparison to normal control; (b) *P* < .05 in comparison to EAC control group.

Catalase activity was evaluated in terms of H_2_O_2_ reduced/min/mg of protein. The catalase level in EAC bearing mice was found to decrease by 57.80% as compared to normal control group (*P* < .001). However, treatment of EAC bearing mice with different doses of ZMS enhanced the catalase activity in a dose-dependent manner (Figure 9). At the highest dose of 800 mg/kg, rise in catalase activity was found to be 44.31% as compared to EAC control group (*P* < .05).

## 4. Discussion

One of the goals of anticancer potential of any drug/extract is the induction of apoptosis in cancer cells [27]. Apoptosis or programmed cell death is one of the most important targets for cancer treatment comprising chemotherapy as well as chemoprevention. It is characterized by membrane blebbing, cytoplasmic condensation, formation of apoptotic bodies, DNA fragmentation, alteration in membrane symmetry, activation of cascade of caspases, and loss of mitochondrial membrane potential [28]. In the present study, cytotoxic potential of ZMS was assessed by MTT assay against cancer cell lines (HL-60, Molt-4, HeLa). Assay is based upon reduction of yellow tetrazolium salt (MTT) by metabolically active cells to a dark blue formazan [29], which has been employed by many workers to measure cytotoxicity to cells [30, 31]. ZMS showed significant cytotoxic effect against different cell lines with minimum IC50 value of 20 *μ*g/mL against HL-60 cells and no significant cytotoxicity to normal cell line was observed. The criteron of cytotoxicity activity for crude extracts established by American National Cancer Institute is an IC50 value of <30 *μ*g/mL in preliminary assay [32], which was observed only against HL-60 cells and hence further investigations were carried out using HL-60 cell line. To investigate the mechanism of cell death induced by ZMS in HL-60 cells, flow cytometric analysis was done by PI and annexin V-FITC labelling to confirm apoptosis/necrosis as a marker to assess apoptosis [33]. During apoptosis, a number of changes occur in cell surface markers that show affinity for PI and annexin V-FITC labelling [34]. The results of the present study indicated an increase in apoptotic population induced by the extract in a concentration-dependent manner. Earlier Malik et al. [35] reported withaferin A as apoptosis inducer utilizing flow cytometric analysis. Another important characteristic of apoptosis induction is DNA fragmentation and measurement of DNA content makes it possible to identify apoptotic cells. To recognize the cell cycle phase specificity and to quantify apoptosis, propidium iodide (PI) dye binds to DNA in cells at all stages of the cell cycle, and the intensity with which a cell nucleus emits fluorescent light is directly proportional to its DNA content. The increase in hypo diploid sub Go population in our results indicates the induction of apoptosis, as sub Go peak is reported to be a quantitative indicator of apoptosis [16]. Uvaretin, Isouvaretin, and Diuvaretin are known triterpenoid established as apoptosis inducer using the same parameters [36]. DNA fragmentation was confirmed by agarose gel electrophoresis. The nuclear DNA of apoptotic cells shows a characteristic laddering pattern of oligonucleosomal fragments, which is regarded as the hallmark of apoptosis [37]. 

Plant extract at various doses was tested *in vivo* against Ehrlich ascite carcinoma induced tumor in Swiss albino mice. Ascite fluid is the direct nutritional source for tumor cells and a rapid increase in ascite fluid with tumor growth would be a mean to meet the nutritional requirement of tumor cells [38]. Treatment with ZMS inhibited tumor volume and viable cell count and also increased the life span of tumor-bearing mice. Prolongation of life span of animal has been well documented as criteria for judging the drug activity [39]. Myelosuppression and anaemia are the major problems of cancer chemotherapy [40]. Treatment with ZMS reverted back the Hb content, RBC, and WBC count towards normalcy showing its protective action on hematopoetic system. Our results are in corroboration with the findings of Gupta et al. [41] who reported a similar effect of *Indigofera aspalathoides* against Ehrlich ascites carcinoma. 

**Figure 10:**
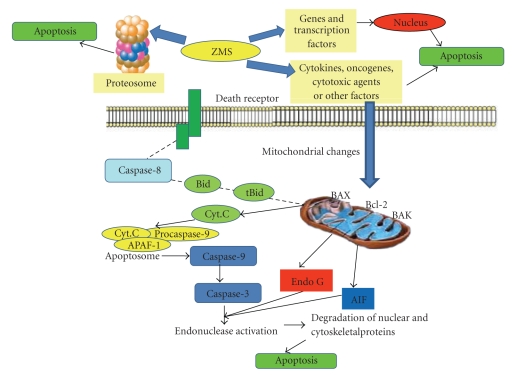
Presumptive mechanism of apoptosis induction by ZMS. ZMS may affect the genes and transcription factors or may affect proteosome to induce apoptosis or may trigger some signals that directly change the mitochondrial permeability transition pore. This induces changes in the members of Bcl-2 family. It may interact with BAX (Bcl-2 associated X protein) and BAK (Bcl-2-proapoptotic member) and stimulate the release of cyt. C (Cytochrome C). Cytochrome C released from mitochondria binds to APAF-1 (Apoptotic protease activating factor-1) and procaspase-9 forming apoptosome and activating caspase-9 which in turn activates executor caspases (Caspase 3, 6, and 7) leading to cell death via apoptosis.

Excessive production of free radicals results in oxidative stress which leads to damage of macromolecules like lipids and induces lipid peroxidation [42, 43]. Malondialdehyde (end product of lipid peroxidation) has been reported to increase in carcinomous tissues than nondiseased organs [44]. Glutathione, a potent inhibitor of neoplastic process is found particularly in liver and known to exhibit protective function against free radicals [42]. In the present study, ZMS treatment reduced lipid peroxidation and enhanced glutathione content in tumor-bearing animals. Superoxide dismutase and catalase are free radical scavenging enzymes present in all oxygen metabolizing cells and provide defence against potentially damaging entities of superoxide and hydrogen peroxide. Inhibition of both the enzymes as a result of tumor growth observed in the present study is in corroboration with earlier reports [45]. Administration of ZMS at different doses increased the SOD and CAT levels in a dose-dependent manner indicating the protective function of ZMS. In our previous study [14], phytochemical analysis of the extract showed the presence of betulinic acid which is a known anticancer agent [11] and the antitumor and apoptosis inducing property of extract may be in part due to the presence of betulinic acid. It is well established that betulinic acid activates apoptosis independent of the death receptors (CD95/Fas), but induces the successive activation of caspase 9 and caspase 3 [46, 47]. The phytochemical analysis of the extract in our previous study has also revealed the presence of alkaloids and flavonoids, which could also be expected to be responsible for its bioactivity. Although at this stage the exact mechanism underlying apoptosis induction by extract is difficult to predict, yet it could be due to activation of mitochondrial pathway or its effect on proteosome or other gene and transcription factors. The hypothetical mechanism is outlined in Figure 10.

In conclusion, the present study highlights the antitumor and cytotoxic potential of seed extract of *Ziziphus mauritiana*. Further studies to characterize the active principles and elucidate the mechanism of action are in progress.

## Figures and Tables

**Table 1:** Effect of ZMS treatment on EAC-bearing mice.

Parameters	Treatment of different groups						
	Control	EAC	EAC + 5FU	EAC + ZMS	EAC + ZMS	EAC + ZMS	EAC + ZMS
	Dose (mg/kg b.wt.)		20	100	200	400	800
Hb content (mg/dl)	13 ± 0.5	9.5 ± 0.73^a^	13.1 ± 0.4^b^	11.24 ± 0.67^b^	11.59 ± 0.6^b^	12.4 ± 0.81^b^	12.2 ± 0.45^b^
WBC (cells/mL × 10^6^)	7.17 ± 0.11	15.6 ± 0.64^a^	7.73 ± 0.68^a^	12.1 ± 0.75^b^	9.4 ± 0.83^b^	9.2 ± 1.26^b^	8.5 ± 0.40^b^
RBC (cells/mL × 10^6^)	5.33 ± 0.45	3.5 ± 0.41^a^	5.2 ± 0.11^b^	4.2 ± 0.55^b^	4.3 ± 0.35^b^	4.9 ± 0.15^b^	5.0 ± 0.12^b^
Tumor Volume (mL)	—	8.3 ± 0.79	0.7 ± 0.20^b^	7.6 ± 0.94^b^	5.4 ± 0.96^b^	4.5 ± 1.04^b^	2.1 ± 0.51^b^
Viable tumor Cell Count (×10^7^)	—	283.02 ± 1.63	11.17 ± 0.96^b^	186.12 ± 1.57^b^	146.27 ± 1.43^b^	109.57 ± 1.08^b^	69.59 ± 1.62^b^
Mean Survival time (Days)	—	20 ± 1.41	39 ± 0.81^b^	23 ± 1.53^b^	26 ± 1.73^b^	30 ± 0.81^b^	34 ± 1.63
Tumor inhibition (%)	—		96.11 ± 0.73	25.67 ± 1.09	39.78 ± 2.55	57.54 ± 1.03	75.42 ± 0.96
Increase in life span (%)	—		95	15	30	50	70

The results are presented as Mean ± S.E.M. (*n* = 10). ^a^ *P* < .001 in comparison to normal group; ^b^ *P*<.05 in comparison to EAC control group.
